# Molecular Interaction of Bone Marrow Adipose Tissue with Energy Metabolism

**DOI:** 10.1007/s40610-018-0096-8

**Published:** 2018-04-28

**Authors:** Karla J. Suchacki, William P. Cawthorn

**Affiliations:** University/British Heart Foundation Centre for Cardiovascular Science, The Queen’s Medical Research Institute, University of Edinburgh, Edinburgh, EH16 4TJ UK

**Keywords:** Bone marrow adipocytes, Bone marrow adipose tissue, Endocrinology and metabolism

## Abstract

**Purpose of Review:**

The last decade has seen a resurgence in the study of bone marrow adipose tissue (BMAT) across diverse fields such as metabolism, haematopoiesis, skeletal biology and cancer. Herein, we review the most recent developments of BMAT research in both humans and rodents, including the distinct nature of BMAT; the autocrine, paracrine and endocrine interactions between BMAT and various tissues, both in physiological and pathological scenarios; how these interactions might impact energy metabolism; and the most recent technological advances to quantify BMAT.

**Recent Findings:**

Though still dwarfed by research into white and brown adipose tissues, BMAT is now recognised as endocrine organ and is attracting increasing attention from biomedical researchers around the globe.

**Summary:**

We are beginning to learn the importance of BMAT both within and beyond the bone, allowing us to better appreciate the role of BMAT in normal physiology and disease.

## Introduction

**Fig. 1:**
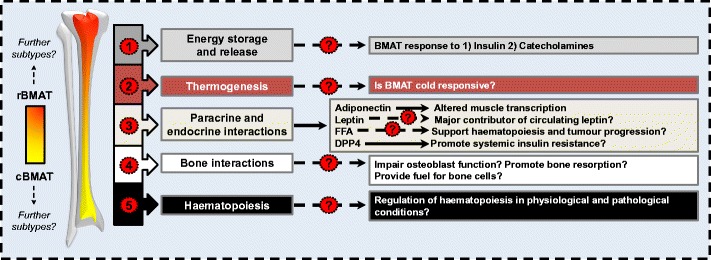
Unknown functions of bone marrow adipose tissue (BMAT)

In humans white adipose tissue (WAT) forms in utero and persists throughout life. Upon formation, adipose tissue is highly active and responds rapidly to external and internal cues [1]. Most commonly, adipose tissue is defined as either WAT or brown adipose tissue (BAT) and is found in discrete and defined locations throughout the body. WAT and BAT depots differ both in their developmental origin and function. WAT stores excess energy as triglycerides and, when needed, catabolises these stores to release fatty acids and glycerol. In contrast, BAT expresses thermogenic proteins (e.g. uncoupling protein 1, UCP1) that allow it to dissipate energy through the production of heat [2]. In addition to WAT and BAT, bone marrow adipose tissue (BMAT) constitutes over 10% of total fat mass in lean, healthy humans. BMAT further increases in diverse clinical conditions, including osteoporosis, ageing, type 2 diabetes, and radiotherapy, with recent studies also showing BMAT regulation in mild spastic cerebral palsy, paediatric non-alcoholic fatty liver disease, inflammatory bowel disease, chronic kidney disease and macrodactyly [3–13]. In stark contrast to WAT, BMAT is also increased in caloric restriction and anorexia nervosa. These observations suggest that BMAT has systemic metabolic actions distinct to those of WAT and BAT. Compared to WAT and BAT, knowledge of BMAT formation and function is extremely limited, despite BMAT being identified over a century ago. This is partly due to the challenges inherent in studying a tissue so diffuse and difficult to access. However, in the last decade BMAT has seen increasing attention among several research fields, including metabolism, haematopoiesis, skeletal biology and cancer. Herein, we review the most recent developments of BMAT research in both humans and rodents, including the distinct nature of BMAT; the autocrine, paracrine and endocrine interactions between BMAT and various tissues, both in physiological and pathological scenarios; how these interactions impact energy metabolism; and the most recent technological advances to quantify bone marrow (BM) adiposity (Fig. 1).

Recent efforts have begun to uncover the role of BMAT in energy metabolism, including insulin responsiveness [14••, 15], the response of BMAT to catecholamines [16•], and potential BAT-like characteristics (see ‘BMAT—a distinct adipose depot?’). However, it remains unclear to what extent BMAT acts as a site of energy storage and release, how paracrine and endocrine actions of BMAT impact energy metabolism, the ability of BMAT to interact with bone cells, and the role of BMAT in haematopoiesis. These topics are covered in the text.

## BMAT—a Distinct Adipose Depot?

The development of BMAT is a normal physiological process that progresses throughout the lifespan, such that by adulthood (25 years) BMAT occupies 50 to 70% of the total bone marrow volume [17]. This increase results from the BM undergoing a red-to-yellow conversion occurring in a centripetal fashion [18, 19]. During normal development this red-to-yellow marrow conversion is independent of WAT accumulation and slows during adulthood. However, in obesity, vertebral BMAT is positively associated with visceral fat [20], suggesting a relationship between BMAT and WAT development in this context. The conversion of red-to-yellow marrow in rodents also occurs in a similar centripetal pattern and, in both rodents and humans, two distinct populations of BM adipocytes have been described: constitutive BMAT (cBMAT) is situated in the distal long bones and (in rodents) caudal vertebrae, while regulated BMAT (rBMAT) is situated in the proximal regions of long bones and in spinal vertebrae [21]. cBMAT and rBMAT are not only anatomically distinct but also show different responses to age, weight, endocrine factors and medical interventions. In brief, cBMAT appears in the early postnatal period, histologically resembles WAT, and mostly does not increase or decrease in response to external stimuli or pathophysiological changes. Conversely rBMAT develops after cBMAT, is interspersed with haematopoietic cells and increases or decreases in various conditions (reviewed [18, 21]). Adipocytes within cBMAT and rBMAT were recently shown to have different metabolic functions, with cBMAT being more resistant to lipolytic stimuli [16•]. Thus, cBMAT and rBMAT may have distinct impacts on metabolic homeostasis.

Beyond this potential heterogeneity between BMAT subtypes, a broader question is whether BMAT function overlaps with that of WAT or BAT. Establishing the pathophysiological roles of BMAT will be crucial to determine whether it represents a new target for disease treatment (reviewed in [22]). Histologically, BMAT adipocytes typically resemble those in WAT; however, whether BMAT is WAT- or BAT-like remains a subject of debate. Indeed, it has been reported that BM has a mixed BAT/WAT population of adipocytes. Krings et al. transcriptionally analysed whole tibiae, revealing detectable expression of BAT markers including *Prdm16*, *Dio2* and *Pgc1α* [23]. Furthermore, they found that aged diabetic mice showed decreased expression of BAT markers in whole tibiae, suggesting that BMAT function changes with ageing and diabetes. Most interestingly, administration of rosiglitazone, a synthetic agonist for adipocyte-specific PPARγ and a potent insulin sensitiser, significantly increased both BAT (*Ucp1*, *Pgc1α*, *Dio2*, *β3AR*, *Prdm16* and *FoxC2*) and WAT (*Adipoq* and *Lep*) markers in whole tibiae of normoglycemic mice, but not in diabetic mice [23]. More recently it has been reported that, compared with cBMAT, rBMAT is characterised by elevated expression of beige fat markers, including *Ucp1* [24]. However, we have shown that *Ucp1* expression in whole tibiae is 10,000- to 25,000-fold lower than in BAT [25]. This level of expression is similar to that observed by Krings et al. and clearly argues against BAT-like functions of BMAT. However, these studies also highlight the technical limitations of using whole bones to represent BMAT. Efficient isolation of a pure population of adipocytes from the bone marrow is technically very difficult, though methods to achieve this have been reported [26]. Advanced imaging approaches are also allowing BMAT-specific analysis, with 3D electron microscopy recently revealing a dense mitochondrial network within BM adipocytes [27].

This latter observation supports the possibility that BM adipocytes may have some brown- or beige-adipocyte-like characteristics. Consistent with this, brown-like adipocytes were observed in a BM core biopsy taken from a 74-year-old man with untreated lymphoplasmacytic lymphoma. The authors reported that these adipocytes were UCP1-positive and largely separated from the surrounding marrow, suggesting that the observed “BAT” may be an incidental intraosseous hibernoma [22, 28]. Furthermore, 18F-FDG-PET/CT imagining of healthy young human subjects, with or without cold stimuli, identified cold-stimulated 18F-FDG uptake in vertebral bone marrow that significantly correlated with 18F-FDG uptake into BAT [29]. The authors further identified expression of *UCP1* and *PRDM16* in BM from two male subjects, while histological assessment of vertebral BM from 3-week-old mice highlighted the presence of multilocular, brown-like adipocytes that were positive for UCP1. Taken together, these data suggest the presence of functional brown adipocytes in vertebral BM of mammals. The authors suggest that the presence of brown adipocytes in the BM seems plausible given that Myf5-positive cells emerge at the juxtaspinal, prospectively paravertebral, regions within somites [30, 31], and it has been well documented that BAT is derived from Myf5-positive myoblastic cells [32].

A recent study provided further evidence for a mixed BAT/WAT phenotype of BMAT both in vitro and in vivo. The authors showed that treatment of BM mesenchymal stromal cells or stromal ST2 cells with triiodothyronine or a thyroid hormone receptor beta-specific agonist (*GC*-*1)* significantly increased expression of brown and beige fat markers. Furthermore, administration of *GC*-*1* in vivo in thyroid hormone-deficient (*Tshr* ^−/−^) mice showed a 149-fold increase in *Ucp1* expression in skeletal tissue (femoral and tibial epiphyses). Whether this translates into detectable UCP1 protein expression remains to be confirmed; however, these data suggest not only that thyroid hormone induces the expression of BAT genes in mouse BMAT but, importantly, that like WAT, BMAT has the potential to beige [33].

Murine tracer models are crucial in search of the origin of BMAT. In contrast with the above data, recent research has shown that the origin of BMAT is likely to be distinct to that of WAT and BAT. WAT is derived from Myf5^−^/Pax7^−^ progenitors, while BAT is derived from Myf5^+^/Pax7^+^ progenitors [14••, 32, 34]. Strikingly, BM adipocytes are also derived from progenitors that express Osterix. Once thought to be osteoblast-specific, Osterix is a transcription factor that acts downstream of Runx2/Cbfa1 and is required for osteoblast differentiation [35]. Lineage tracing studies using Prx1-Cre:mT/mG and Osx1-Cre:mT/mG showed that tibial and femoral BM adipocytes were traced in Prx1-Cre:mT/mG mice as well as endosteum osteocytes, articular chondrocytes and inguinal WAT. Furthermore, in Osx1-Cre:mT/mG mice, tracing occurred only in adipocytes in BMAT, and not those in WAT or BAT depots, suggesting that BMAT arises from a mesenchymal-osteogenic lineage [36–38] (Comprehensively reviewed in [14••]). Further unpublished lineage tracing studies have shown that insulin signalling is not required for lipid accumulation in BMAT, but is required in WAT, providing evidence for the functional differences in regulation of adipose depots [14••].

## BMAT Autocrine, Paracrine and Endocrine Interactions

### Sexual Dimorphism

The distribution of WAT and BAT shows a clear sexual dimorphism [39, 40] that seems to also apply to BMAT. We have found that female mice tend to have a higher volume of rBMAT in the proximal tibial diaphysis [41], whereas in humans BM fat is higher in males than in females, at least in younger adults [42]. To further explore such sex differences, Lecka-Czernik et al. investgiated the impact of sex steroids on BMAT [24]. While no sexual divergence in the volume and transcriptional profile of distal BMAT was observed, ovariectomy led to increased BMAT, increased *Adipoq* (adiponectin) and decreased beige fat gene markers. Orchidectomy in males also tended to increase BMAT, consistent with previous observations that testosterone can decrease BMAT in female rats [43]. However, unlike ovariectomy, orchidectomy did not change expression of *Adipoq* or beige fat gene markers [24]. Thus, males and females may differ in how their sex steroid deficiency affects BMAT formation and endocrine function [24].

In humans, the impact of testosterone on BMAT remains to be established, but the effects of oestrogen are similar to those observed in rodent studies. For example, Limonard et al. used MRI to measure vertebral BM fat in postmenopausal women before, during, and following a two-week treatment with 17-β estradiol. Even in such a short timescale, this treatment was found to rapidly decrease the BM fat fraction [44]. Consistent with this, in younger adults vertebral BM fat tends to be higher in males than in females, whereas, post-menopause, females have greater vertebral BM fat content [42]. Thus, oestrogen clearly has a profound ability to suppress BMAT formation, which likely contributes to the sex differences in BMAT content across the lifecourse. More recent studies suggest that BMAT is also regulated by follicle-stimulating hormone (FSH). Specifically, mice that underwent ovariectomy and were then given an FSH antibody had decreased BMAT volume coupled with decreased fat mass and the production of UCP1-positive adipose tissue [45]. Thus, beyond oestrogen and testosterone, it will be important to determine if other sex hormones can also regulate BMAT formation and function in humans.

### Adiponectin

BMAT is known to increase in caloric restriction (CR), contributing to increases in circulating adiponectin [46, 47]. Adiponectin is the most abundant adipokine in the circulation and can be used as a clinical biomarker for early detection of conditions such as type 2 diabetes, cardiovascular diseases, and certain cancers. Although we have recently reviewed this elsewhere [47], many questions remain regarding adiponectin secretion from BMAT and how this is altered by disease. For example, recent human data suggest that adiponectin may regulate the relationship between BMAT and insulin sensitivity in obese and non-obese premenopausal women [48]. It remains unclear if adiponectin secretion from BMAT directly influences cardiac function or the risk of cardiovascular disease. Studies in rats showed that bone marrow and adipose mesenchymal stem cells attenuated cardiac fibrosis [49]. However, far more research effort is required to understand if BMAT directly impacts cardiac function or impacts other tissues, such as the liver and pancreas, that are known to be targeted by adiponectin [50].

### Bone Trauma and Pathology

Given the close proximity of BMAT to the bone, researchers have suggested that BMAT directly regulates bone metabolism. In the last decade, bone has emerged as a highly metabolic organ that contributes to the regulation of whole-body metabolism [51]. Indeed, bone has the capability to regenerate without scar formation following mechanical and structural failure. This occurs typically by secondary endochondral healing, which consists of both endochondral and intramembranous ossification. The process of secondary endochondral healing begins with haematoma formation; then an acute inflammatory response with pro-inflammatory signalling; primary callus formation (soft callus), which undergoes revascularisation and calcification to form the hard callus; and finally bone remodelling [52]. This process of fracture healing is highly metabolically demanding and requires the cooperation between different cell types, as recently reviewed elsewhere [53]. BM adipocytes secrete adipokines (e.g. adiponectin), cytokines (e.g. RANKL), and free fatty acids that act to promote bone resorption, haematopoietic recovery and supply energy. Indeed, BMAT has been reported to be increased in subjects with prevalent vertebral fracture [54, 55] and prevalent vertebral deformities [56]. Despite recent efforts, it remains unclear why BMAT increases in fracture and, specifically, why there is an intricate relationship between bone and BMAT during bone pathology. Moreover, in cases of failed bone regeneration, such as non-union fracture, it remains unclear if BMAT has a positive or negative impact on bone healing [57]. Most recently, Ambrosi et al. elegantly showed that BMAT exerts direct negative effects on bone healing and haematopoiesis in mouse models, with BM adipocytes secreting dipeptidyl peptidase-4 (DPP4) to inhibit fracture repair [58]. Given that DPP4 is a target of anti-diabetic therapies, this finding raises the intriguing possibility that BMAT-derived DPP4 might impact not only skeletal remodelling, but also systemic insulin resistance.

Osteoarthritis (OA) is a heterogeneous disease that leads to the progressive loss of normal joint function and related subchondral bone changes. OA is the most common form of arthritis and represents the world’s leading cause of physical disability in adults. At a time when the population is ageing and the prevalence of obesity is increasing, there has never been a greater need for the prevention of OA. Currently, OA is routinely managed therapeutically and patients are offered physiotherapy [59]. Traditionally, OA was considered to be a disease of the cartilage, but more recently has been shown to affect the entire joint, including the bone, synovium, tendons, and muscles [60]. The location of BMAT suggests that BM adipocytes may play a role in the pathology of OA. A recent study found increased BMAT in postmenopausal women with both OA and osteoporosis (OP), and the authors suggested that a subgroup of OA subjects with elevated BMAT may have a high risk of developing OP [61].

### Interaction of BMAT with Bone Cells

BMAT has recently been shown to be responsive to parathyroid hormone and to secrete receptor activator of nuclear factor kappa-Β ligand (RANKL), a key regulator of osteoclast differentiation and activation [26]. This suggests that BMAT exhibits unique osteo-resorptive characteristics. Furthermore, murine models with decreased sclerostin (SOST) have significantly decreased BMAT [62], suggesting that BMAT formation is governed directly by osteocytes, which secrete SOST. Currently, there is much interest in anti-SOST therapy for treatment of OP. This is driving a greater consideration of the clinical implications of BMAT, which should help to further our knowledge of this complex adipose depot [62, 63]. In murine models and human patients with low bone mineral density (BMD) and bone formation, BMAT has been shown to be elevated (reviewed [64••, 65]). Mice with a loss of function mutation in the *Dock7* gene, which results in low BMD and reduced bone formation, have very few osteoblasts but BMAT is increased 3.5-fold compared to wild-type controls. However, it is important to highlight that other studies have not found an inverse relationship between BMD and BMAT. For example, BMAT was significantly increased following 12 weeks of high-fat diet feeding in mice, while BMD was unchanged [66]. Furthermore, C3H/HeJ mice have increased bone mass and BMAT compared to C57BL/6 mice [67], and we have shown that during CR in rabbits, bone loss can occur independently of BMAT expansion [41].

### Metastasis, Myeloma and BMAT

The interplay between tumour cells, osteoclasts and osteoblasts is well documented, but despite occupying 50 to 70% of the total BM volume, the relationship between BMAT, multiple myeloma, and metastasis of breast and prostate has only just begun to be understood. While beyond the scope of this review, others have recently provided elegant overviews of the growing evidence linking BMAT to tumour growth and the development of associated bone disease [68–71].

## Technological Advances to Assess BMAT Quantity and Metabolic Function

As reviewed elsewhere in this issue, techniques for BMAT imaging are improving and expanding. Standard histological approaches, such as haematoxylin and eosin staining of paraffin-embedded sections, have been used routinely to detect adipocytes within BM. More recently, osmium tetroxide staining followed by micro-computed tomography (μCT) has been used to visualise and quantify BMAT in situ [72]. New μCT contrast agents are currently being developed to assess BMAT, vasculature and nerves without the requirement for time-consuming decalcification. The most recent of these stains, for use in contrast-enhanced microfocus computed tomography (CE-CT), is a Hafnium-based Wells-Dawson polyoxometalate (Hf-POM) [73, 74]. Hf-POM has been validated in murine long bones and provides the researcher with a 3D representation of the mineralised bone, vasculature and BMAT. These developments in contrast agents allow novel insights into the interaction between the bone, vasculature and BMAT. However, as for osmium μCT, specialist high-resolution CT scanners are required*.*

One relatively simple technique has been developed by the Horowitz laboratory, allowing BMAT to be visualised ex vivo by confocal microscopy (described in [14••]). Briefly, the BMAT is extracted from long bones by using a needle to punch through the length of the diaphysis. This BMAT plug is then gently ejected onto a slide, immersed in Fluoromount-G (eBioscience), cover-slipped and then visualised. This method does not affect intrinsic fluorescence (e.g. mT/mG reporter mice) and allows staining of neutral lipids within the BM adipocytes.

Studies in human subjects routinely use CT and magnetic resonance spectroscopy (MRS) and imaging (MRI) methods to measure BMAT. A comprehensive review of these techniques can be found elsewhere in this issue; however, it is worth highlighting the developments in BMAT imaging that are particularly relevant to its metabolic characteristics and functions. For example, ex vivo high-resolution magic angle spinning (HRMAS) proton nuclear magnetic resonance (^1^H NMR) spectroscopy has recently been used in humans to determine the (un)saturation level of fatty acids in BMAT [75]. A novel, dual-energy computed tomography (DECT) method has also been described to assess both BMAT and marrow-corrected volumetric BMD (mcvBMD) [76]. Hopefully, these developments in new clinical techniques will allow researchers to more accurately assess BMAT’s lipid composition and relationship with skeletal parameters.

Finally, imaging studies are beginning to shed light on the metabolic functions of BMAT. Positron emission tomography–computed tomography (PETCT) is a non-invasive technique allowing the visualisation of biological processes. Most simply, compounds are radiolabelled with positron-emitting radioisotopes (e.g. ^11^C, ^15^O, and ^18^F). These radiolabelled compounds emit positrons which annihilate with electrons and produce two annihilation photons that travel in opposite directions and are detected by the PET scanner. Upon detection, the PETCT scanner uses various algorithms and attenuation correction factors to hybridise the PET data with the CT images, allowing the user to visualise and analyse the anatomical locations of the tracer. PET imaging can be conducted either dynamically or statically. Clinically, static imaging is most common as dynamic analysis requires kinetic modelling, which is complex and time consuming [77]. The most commonly used radiotracer is ^18^F-fluorodeoxyglucoe (^18^F-FDG), a glucose analogue that is actively transported into the cell by glucose transporters. After import, ^18^F-FDG can be phosphorylated by hexokinase to form 2-deoxyglucose-6-phosphate; however, unlike endogenous glucose, once phosphorylated ^18^F-FDG is unable to continue along the glycolytic pathway and instead accumulates in the cell as ^18^F-labelled 2-deoxyglucose-6-phosphate. This intracellular accumulation of ^18^F allows the user to calculate the standardised uptake value (SUV, the concentration of radioactivity in a specific anatomic region, normalised to body weight and the injected dose of the radiotracer), which allows for the quantification of glucose utilisation, commonly in sites of inflammation, tissue repair and cancer [78]. One study in humans assessed ^18^F-FDG uptake into femoral and vertebral BM during a hyperinsulinaemic-euglycaemic clamp, the gold-standard in assessing insulin sensitivity [15]. This revealed that insulin sensitivity in femoral BM, but not in vertebral BM, correlates with whole-body insulin sensitivity and is improved by exercise. However, it is unclear if this reflects insulin action in BMAT or in other BM components. Indeed, in another study this group used ^18^F-FDG PET/CT, combined with MRI analysis of BMAT, to further characterise BMAT metabolic functions. They found that vertebral BM uptake was inversely associated with BMAT content in both diabetic and healthy pigs [79]. This suggests that BMAT itself may not be highly insulin responsive. In addition to ^18^F-FDG, other tracers that target, for example, translocator protein 18 kDa (TSPO) may be useful in dissecting BMAT function. TSPO is an outer mitochondrial membrane transporter that is downregulated in both WAT and BAT during obesity [80] and can be used to image human BAT mass under thermoneutral conditions [81]. Therefore, TSPO may be useful to further understand the phenotypic WAT/BAT properties of BMAT. Clearly, PET/CT holds great potential to improve our understanding of the metabolic functions of BMAT and their contribution to systemic energy homeostasis.

## Conclusions

Though still dwarfed by research into WAT and BAT, BMAT is attracting increasing attention from biomedical researchers around the globe. Indeed, 2017 saw the creation of the International Bone Marrow Adiposity Society (http://bma-society.org/), which aims to further our knowledge of this intriguing tissue. Thus, we are beginning to learn about the importance of BMAT both within and beyond the bone, allowing us to better appreciate the role of BMAT in normal physiology and disease. These advances will hopefully allow us to develop and explore new therapeutic agents to treat bone disease, metabolic disease and skeletal tumours, including both haematological malignancies and metastases from elsewhere. In order for this encouraging progression to continue, new murine models must be created to determine (1) if the physiological location of BMAT affects its physiological role; (2) if different populations of adipocytes are present within BMAT; and (3) if the role of BMAT changes during ageing and disease. In addition, as methods for clinical BMAT measurement become better established, it will be important to apply these more routinely so that BMAT content can be assessed across broader populations. Such information should help to better resolve the physiological and pathological functions of this relatively neglected adipose subtype. Ultimately, now is a very exciting time to be studying BMAT: while there is much more still to learn, research in this field is burgeoning. Thus, we look forward to many key discoveries in the years ahead.
